# 非小细胞肺癌脑转移靶向治疗研究进展

**DOI:** 10.3779/j.issn.1009-3419.2014.11.09

**Published:** 2014-11-20

**Authors:** 涛 蒋, 彩存 周

**Affiliations:** 200065 上海，上海市肺科医院肿瘤科 Deportment of Oncology, Shanghai Pulmonary Hospital, Shanghai 200065, China

**Keywords:** 肺肿瘤, 脑转移, 靶向治疗, Lung neoplasms, Brain metastases, Targeted treatment

## Abstract

肺癌最常见的远处转移部位之一是脑部，肺癌脑转移的发生率为20%-65%，是脑转移性肿瘤中最常见的类型，同时也是肺癌死亡率高居不下的原因之一。分子靶向治疗已成为肺癌治疗的重要手段，分子靶向药物也成为继全脑放疗、立体定向放疗和化疗之后肺癌脑转移新的治疗方法。目前，对脑转移的非小细胞肺癌患者应用靶向药物治疗的研究也越来越多，其安全性和有效性是研究的重点。本文就靶向药物治疗非小细胞肺癌脑转移的研究进展做一综述。

## Abstract (en)

Lung cancer is characterized by the highest incidence of solid tumor-related brain metastases, which are reported the incidence ranged 20% to 65%. This is also one of the reasons why it can cause significant mortality. Molecular targeted therapy plays a major role in the management of brain metastases in lung cancer. Targeted agents have become the novel methods for the treatment of lung cancer with brain metastases beyond the whole brain radiation therapy, stereotactic radiosurgery and chemotherapy. Recently, more and more studies and trials laid emphasis on the targeted agents for non-small cell lung cancer (NSCLC) brain metastases treatment. The key point is the efficacy and safety. In this paper, the targeted treatments of NSCLC brain metastases were summarized.

## 1 概述

肺癌是我国发病率最高的恶性肿瘤之一，《2013年中国肿瘤登记年报》显示：“肺癌已经成为我国恶性肿瘤发病的第一位，每年新发病例达60万。”肺癌最常见的远处转移部位之一是脑部，肺癌脑转移的发生率为20%-65%^[1, 2]^，是脑转移性肿瘤中最常见的类型。曾有报道^[3, 4]^指出，大约有40%确诊肺癌的患者在疾病进程中出现脑转移。其中非小细胞肺癌（non-small cell lung cancer, NSCLC）患者占据了大部分。NSCLC合并脑转移的患者预后很差，有关报道^[5]^指出其中位生存期为7个月，1年生存率仅为20%。

在过去的几十年间，对于NSCLC脑转移的患者，治疗方法主要包括以下几种：①全脑放疗（whole-brain radiotherapy, WBRT）。总体来说，WBRT可以快速缓解神经症状，提高患者生存期。但是一项Ⅲ期随机临床试验^[6]^在比较最佳支持治疗（optimal supportive care, OSC）联合WBRT和单纯OSC对NSCLC脑转移患者的治疗效果时发现，两组的中位生存期分别为49 d、51 d，差异无统计学意义（*P*＞0.05），且生存质量无明显差异。②立体定向放射治疗（stereotactic radiosurgery, SRS）。立体定向放射治疗具有定位精确、剂量集中和安全快速等特点，并且具有非侵袭性、损伤相对较小和恢复快等优点，可以快速控制局部肿瘤进展，缓解神经系统症状。但Kim等^[7]^比较了分别以化疗，WBRT和SRS作为NSCLC无临床症状的脑转移患者的主要治疗手段的疗效后发现，三组间的中位生存期分别为13.9个月、17.7个月、22.4个月，差异无统计学意义（*P*=0.86）。③手术治疗。目前手术治疗在NSCLC脑转移的患者中应用十分有限，仅局限于下列患者：颅内为孤立性病灶或相互靠近的多个病灶；病灶位置较表浅、位于非重要功能区；患者的全身状态良好；肺部病灶已控制，无其他器官转移。而对于大多数NSCLC脑转移的患者而言，在明确诊断时，就已经失去手术治疗的机会了。④化疗。化疗在NSCLC脑转移患者的治疗中占有很重要的位置。传统观点认为化疗药物难以通过血脑屏障，对脑转移的治疗效果有限。但近期研究表明肺癌发生脑转移时血脑屏障已有破坏，存在一定的通透性。许多化疗药物可以透过血脑屏障进入脑内，发挥抗肿瘤作用。许多临床试验^[8-11]^已经证实：以铂类药物为基础，联合培美曲塞、长春瑞滨等化疗药物可使NSCLC脑转移患者生存获益，对颅内病灶的有效率较高，中位生存期可达7.4个月-9.1个月，毒副反应可以耐受。

近年，随着分子生物学的发展，分子靶向治疗已成为NSCLC治疗的重要手段，分子靶向药物也为NSCLC脑转移的治疗提供了新的方法。目前，对脑转移的NSCLC患者应用靶向药物治疗的研究越来越多，其安全性和有效性是研究的重点。本文就靶向药物治疗NSCLC脑转移的研究进展作一综述。

## 2 表皮生长因子受体酪氨酸激酶抑制剂（epidermal growth factor receptor tyrosine kinase inhibitor, EGFR-TKI）

### 2.1 EGFR-TKI单药治疗

EGFR-TKI目前已广泛用于NSCLC的治疗，其中女性、不吸烟、东亚人种及*EGFR*突变患者经EGFR-TKI治疗效果较好。IPASS、NEJ002、WJTOG3405、EURTAC、OPTIMAL等研究已证实EGFR-TKI一线治疗*EGFR*突变的晚期NSCLC患者的疗效优于化疗。EGFR-TKI对*EGFR*突变型的NSCLC患者治疗的缓解率（response rate, RR）可达60%-80%，中位无进展生存期（progression free survival, PFS）可达8个月-13个月，且毒副作用小，与化疗相比可以明显改善患者的生活质量^[12-14]^。据Weber等^[15]^的研究报道：EGFR-TKI能通过血脑屏障，可用于NSCLC脑转移患者的治疗，其代表性的药物有吉非替尼和厄洛替尼。对于*EGFR*突变型的NSCLC脑转移患者，EGFR-TKI治疗的反应率可达60%-100%，中位总生存期（overall survival, OS）可达15个月-20个月，无进展生存期可达6.6个月-11.7个月（表 1）^[16-24]^。尽管目前还没有足够的证据来说明EGFR-TKI中哪一种药物具有更好的疗效，但已经报道的研究提示，在相同的推荐剂量下，厄洛替尼比吉非替尼具有更高的血药浓度，且其具有更高的血脑屏障透过比例，并能向颅内转移灶靶向浓聚等优点。这提示我们厄洛替尼可能使NSCLC脑转移的患者获得更大的治疗效益。

**1:** EGFR-TKIs治疗NSCLC脑转移患者的临床试验和研究 Trials and studies about the treatment of EGFR-TKIs for NSCLC patients with brain metastases

Author	Year	N	Pts selection	Prior treatment	Treatment	ORR (%)	DCR (%)	PFS (mo)	OS (mo)
Ceresoli *et al*^[16]^	2004	41	Unknown	Chemotherapy or WBRT	Gefitinib	10.0	27.0	3.0	5.0
Hotta *et al*^[17]^	2004	14	Unknown	No	Gefitinib	42.9	100	7.7	9.9
Namba *et al*^[18]^	2004	15	Unknown	No	Gefitinib	60.0	67.7	NA	8.3
Kim *et al*^[19]^	2009	23	No smoker	No	Gefitinib	69.6	82.6	7.1	18.8
Porta *et al*^[20]^	2011	17	*EGFR* mutated	No	Erlotinib	82.4	90.0	11.7	12.9
Park *et al*^[21]^	2012	28	*EGFR* mutated	No	Erlotinb	83.0	93.0	6.6	15.9
Welsh *et al*^[24]^	2013	40	Unknown	WBRT	Erlotinib	86.0	NA	9.3	11.8
Iuchi *et al*^[22]^	2013	41	*EGFR* mutated	No	Gefitinib	87.8	NA	14.5	21.9
Song *et al*^[23]^	2014	103	*EGFR* mutated	Radiotherapy	Gefitinib	11.7	53.4	9.0	NA
N: number; Pts: patients; ORR: objective response rate; DCR: disease control rate; PFS: progression free survival; OS: overall survival; WBRT: whole brain radiotherapy; NA: not available; EGFR-TKI: epidermal growth factor receptor tyrosine kinase inhibitor; NSCLC: non-small cel l lung cancer.									

### 2.2 EGFR-TKI序贯或联合疗法

临床上EGFR-TKI不仅可以作为单药使用，也经常联合其他治疗方法。一项观察厄洛替尼联合WBRT治疗NSCLC脑转移患者疗效的Ⅱ期临床研究^[24]^，入组的40例患者经治疗后，总疾病控制率为86%，中位生存期11.8个月；其中17例明确*EGFR*突变状态的患者中，*EGFR*突变型和EGFR野生型的中位生存期分别为19.1个月和9.3个月，整个治疗中没有增加神经系统的毒性。说明EGFR-TKI联合WBRT较单纯WBRT或许可以提高疗效。然而最新的一项Ⅲ期临床研究实验（RTOG 0320）^[25]^，在比较了WBRT+SRS、WBRT+SRS+替莫唑胺、WBRT+SRS+厄洛替尼三种治疗方案后发现：WBRT+SRS联合厄洛替尼或替莫唑胺治疗方案缩短了患者的生存期，并且增加了毒副反应。NSCLC伴1个-3个脑转移灶的126例患者入组，随机分为WBRT+SRS组（44例）、WBRT+SRS+替莫唑胺组（39例）、WBRT+SRS+厄洛替尼组（41例），三组的中位生存期分别为13.4个月、6.3个月、6.1个月，WBRT+SRS组的生存期较其他两组明显延长（*P*＜0.01），6个月内WBRT+SRS组的中枢神经系统症状进展率和生存质量较其他两组有明显优势（*P*＜0.01），三组的3级-5级毒副反应率分别为11%、41%、49%（*P*＜0.01）。其他的Ⅱ期临床试验则报道EGFR-TKI联合WBRT是安全有效的（表 1）。但由于这些研究样本量较小、混杂因素较多，结论还有待设计更加完善的多中心、大样本的临床试验来考证。

## 3 棘皮动物微管相关蛋白样4间变性淋巴瘤激酶融合基因抑制剂（echinoderm microtubule associated protein like 4 anaplastic lymphoma kinase inhibitor, EML4-ALK inhibitor）

EML4-ALK是NSCLC中新发现的癌变驱动基因^[26]^。在NSCLC患者中有约3%-7%的患者含有该融合基因，一般不与*EGFR*、*K*-*ras*、*HER2*及*BRAF*突变同时存在^[27]^。ALK抑制剂的代表药物是克唑替尼。目前关于克唑替尼用于治疗NSCLC脑转移的临床资料还很少^[28]^，但现有的一项单臂Ⅱ期临床试验（PROFILE 1005）和一项随机Ⅲ期临床试验（PROFILE 1007）在分析了入组的存在脑转移的111例患者后发现：克唑替尼治疗NSCLC脑转移的缓解率为9%，疾病控制率为79%。在PROFILE 1007试验中，40%的NSCLC脑转移的患者存在*ALK*基因重排。这些数据提示我们，克唑替尼有可能对成为*ALK*融合基因（ALK+）的NSCLC脑转移患者新的治疗手段。但也有报道^[29]^称克唑替尼血脑屏障透过率很低，无法在脑脊液中达到有效治疗浓度。以及克唑替尼的耐药性问题和其对于ALK野生型的患者治疗无益。这些问题已经引起了人们的足够重视，目前正在研制开发的第二代ALK抑制剂有可能解决上述问题，并为ALK+ NSCLC脑转移的患者带来新的曙光。

## 4 血管内皮生长因子（vascular endothelial growth foctor, VEGF）拮抗剂

VEGF在肿瘤新生血管形成过程中起到了重要的作用，其代表药物主要有阿瓦斯汀和贝伐珠单抗。贝伐珠单抗与铂类、紫杉醇类药物联用可以有效改善非鳞癌NSCLC患者的预后。以往的临床试验往往会把合并脑转移作为NSCLC患者排除入组的标准，认为他们会增加颅内出血的比率。但Socinski等^[30]^在回顾的了17项关于贝伐珠单抗的临床试验后发现，贝伐单抗治疗NSCLC脑转移的患者是安全有效的，并且不会增加中枢神经系统出血的发生率。一项纳入了91例的二期临床试验，评估贝伐珠单抗联合卡铂，紫杉醇作为一线治疗方案，联合厄洛替尼作为二线治疗方案的安全性和有效性，结果一线治疗组颅内病灶缓解率为64.2%，二线治疗组颅内病灶缓解率为20.8%，二者均高于对照组；随访过程中仅有1例出现颅内出血（Grade 1）。这些数据提示我们，VEGF拮抗剂和其它靶向药物的联用，可能会使NSCLC脑转移患者的治疗获益。

## 5 其他靶向抑制剂

目前我们还没有见到关于K-ras、HER-2、BRAF、RET等的抑制剂用于治疗NSCLC脑转移的报道，但随着我们对NSCLC脑转移相关分子机制的深入研究，上述靶向抑制剂将可能用于NSCLC脑转移部分亚型患者的治疗。

## 6 *EGFR*突变与NSCLC脑转移

*EGFR*突变不仅在肺腺癌的患者非常常见，在NSCLC脑转移的患者，也发现了*EGFR*的突变。Matsumoto等^[31]^研究发现，在肺腺癌脑转移患者的标本中发现*EGFR*突变状态可能在肺癌早期的发生发展和脑部转移起到重要作用。一项回顾性研究分析了110例进展期NSCLC患者的原发病灶的*EGFR*突变情况，结果发现晚期出现脑转移的患者*EGFR*突变频率明显高于没有出现脑转移的患者。一项由纪念斯隆-凯瑟琳癌症中心主持的纳入139例NSCLC脑转移患者的随访研究^[32]^，结果显示：使用EGFR-TKI的患者5年生存率为60%，而没有使用的患者仅为20%-30%。然而，一项纳入100例明确*EGFR*突变的NSCLC患者在使用了EGFR-TKI之后增加了患者出现脑部转移的风险^[33]^。总之，目前*EGFR*突变与NSCLC之间的关系还没有明确的定论，EGFR-TKI能否给NSCLC脑转移的患者带来生存获益需要更多的临床证据来说明。

## 7 展望

肺癌脑转移的发生率较高^[1, 2, 34]^，系统治疗是目前NSCLC脑转移患者的主要治疗方法，化疗和靶向治疗各有其特点。有关学者建议将两者联合应用于NSCLC脑转移患者的治疗。但如何确定二者的治疗顺序，何时采用化疗或靶向治疗可以使获得最大的生存效益，仍需要更多的临床试验证据。NSCLC脑转移的靶向治疗已经被越来越多的试验证实安全有效。在考虑使用靶向药物时，最重要的是要筛选相应亚型的患者人群。另外，对于NSCLC脑转移分子机制的深入研究将有助于我们对其的早发现、早诊断、早治疗，最终达到提高患者生存率、改善预后的目标。
